# Near Zero Index Perfect Metasurface Absorber using Inverted Conformal Mapping

**DOI:** 10.1038/s41598-020-66476-x

**Published:** 2020-06-16

**Authors:** Dwight W. Swett

**Affiliations:** Aramco Americas, Aramco Research Center, 17155 Park Row Drive, Houston, Texas 77084 United States

**Keywords:** Engineering, Materials science, Physics

## Abstract

The geometry of the characteristic element forming the artificial structure of an electromagnetic metamaterial defines the way the metamaterial will interact with electromagnetic waves, and accordingly, how it will transmit, reflect, and absorb electromagnetic energy. Metamaterials have been discovered that can manipulate electromagnetic waves to create perfect absorption of incident electromagnetic energy using relatively simple elemental geometries. But the phenomenon is confined to very narrow frequency bandwidths owing to the mono-resonance characteristics of simple cellular structures. Complex cellular geometries based on the combination of many different fundamental building blocks may be able to constructively couple many more resonances and broaden the perfect absorption bandwidth. We describe here a metasurface based upon geometric inversion of a set of conformal mapping contours. The resulting geometry forms a nearly continuous series of perfect absorption resonances within an ultrathin (*λ*/165) metasurface to develop broadband absorption in a frequency range of interest for downhole chemical spectroscopy. The metasurface is derived from a geometric inversion of the Rhodonea, or more commonly called four-leaf roses, conformal mapping contours and was found to exhibit a near zero index metamaterial (NZIM) behavior. An uncooled microbolometer design is described that uses the metasurface geometry on a single VO_2_ thermometric substrate leading to an infrared detector with predicted maximum absorption of 99.94% at 4.3 *μ*m and an absorption bandwidth of 170% FWHM on 15.8 *μ*m center wavelength, coincident with important chemical spectra of downhole hydrocarbons. The infrared detector design has a predicted maximum detectivity D* = 1.5 × 10^9^ cm$\sqrt{\text{Hz}}$/W and noise equivalent difference temperature NEDT of 70 mK at a frame rate of 60 Hz. These levels of detector performance conventionally would be achievable only with cryogenically cooled technologies and could represent a significant step in the effort towards deploying an in situ infrared chemical spectroscopy sensor into downhole logging applications.

## Introduction

Electromagnetic metamaterials are artificial composite materials that exhibit unusual electrical and magnetic properties derived from the resonance behavior of subwavelength electrically conductive elements. Many of these unusual electromagnetic properties are not found in naturally occurring materials, hence the use of the term “meta”, or outside of nature. The theory of electromagnetic metamaterials suggests that arbitrary effective properties can be developed at any specific frequency, conceivably by simply manipulating the design of special subwavelength resonator elements. This has led to a wide range of theoretical and practical designs for metamaterials with unlikley physical properties such as simutaneously negative electrical permittivity and magnetic permeability corresponding to negative refractive index, evanescent wave enhancement, antiparallel wave propagation and energy flow, and zero index materials. Electromagnetic metamaterials derive their unusual properties from the geometric structures that comprise the composite rather than the materials themselves. Creation of the desired properties in a metamaterial is then a matter of development of the appropriate geometric elements for the frequency range(s) of interest. This concept of arbitrarily tailorable electromagnetic response is compelling to consider indeed, particularly with respect to the development of infrared sensor technologies which have historically been limited by the electromagnetic absorption properties of natural materials. One of the approaches for infrared sensing is based upon thermal detection using arrays of very small thermal mass detector elements in order to interact with one or more electromagnetic modes. Usually these are applied as broadband devices that image spectrally near-uniform objects, but other applications such as chemical detection rely upon the broadband characteristics of thermal detectors in spectroscopic analysis using interferometric methods. Current thermal imaging technologies have limited detectivity unless deployed with active cooling, which makes them unrealistic for downhole chemical analysis applications. Electromagnetic metamaterials may offer a solution that migrates uncooled microbolometer technologies into the detector performance regime currently occupied by cryogenically cooled systems.

Fundamental research is being undertaken to understand how the unusual effective material properties of metamaterials affects such basic phenomena as diffraction, absorption, and emission. N. Liu *et al*.^1^ investigated a narrowband infrared perfect metamaterial absorber and its application as a plasmonic sensor. They experimentally demonstrated a narrowband perfect plasmonic absorber at *λ* = 1.6 *μ*m with polarization-independent absorption of 99% at normal incidence and more than 90% absorption over a wide angular range of incidence between +/−80 degrees. They showed the frequency shift in the minimum of the reflectance spectrum to be extremely sensitive to the refractive index of the media in which the metamaterial composite is immersed, thereby forming a plasmonic refractive index sensor. Chu *et al*.^2^ utilized the concept of metasurfaces, ultrathin metamaterial films, to design and experimetally validate an invisibility cloak that functions in transmission geometry as opposed to reflection geometry using conventional approaches based upon transformation optics. This distinction gives the illusion of transparency to a cloaked object. Rana *et al*.^3^ developed a Tungsten-based solar energy absorber based upon metasurfaces which is predicted to achieve practically 100% absorption in the visible range of 400–800 nm. The Tungsten based design is much better able to accomodate high temperatures owing to the high melting point (3422 °C). Other researchers have investigated readily applicable solutions using metamaterials for new infrared and terahertz microbolometer technologies^4–7^ with measurable success in regard to detectivity enhancement and noise reduction. The common objective was to increase electromagnetic energy absorption without incurring a thermal mass penalty thereby improving detectivity. Current results in the literature would suggest that incorporation of known metamaterial absorber designs has advanced uncooled technologies to approach detectivities ${\text{D}}^{\ast }=3.0\times {10}^{8}\,\text{cm}\sqrt{\text{Hz}}/\text{W}$ and noise equivalent difference temperature NEDT of 100 mK or less, at frame rates between 10–30 Hz.

Historically, gas chromatography has been utilized extensively for fingerprinting and analyzing reservoir fluids in the evaluation of reservoir compartmentalization and connectivity (Larter *et al*.^8^, and Permanyer *et al*.^9^). These analyses are typically performed at the surface or off-site using samples retrieved from a variety of open-hole formation sampling and testing services or later from the production flow. Currently however, gas chromatography is far from adaptable to downhole application and is effectively confined to the laboratory. An alternative approach, using the electromagnetic resonance characteristics of the fluid, is based upon infrared spectroscopy. Infrared spectroscopy relies on the fact that electromagnetic absorption causes changes in the vibrational and rotational motion of the molecules being irradiated. These molecular vibration changes result in spectral bands of absorption that are dependent upon the vibrational resonance frequencies of the molecules, while the absorption intensities depend on how well the energy couples to the molecular resonances.

All compounds except for elemental diatomic gases (such as N_2_, H_2_ and O_2_) have infrared spectral resonances, implying that conceptually all the components of interest for hydrocarbon production from downhole fluids could be analyzed using their characteristic infrared absorption bands. A Fourier transform infrared (FTIR) spectroscopy instrument would be the preferred choice for an *in situ* downhole application due to the high modulation frequencies that can be achieved with the method. In the oil and gas industry FTIR spectroscopy has been applied in combination with chemometrics and statistical data analysis to achieve rapid rig site chemical analysis of crude oils (Abdulkadir *et al*.^10^, Aske *et al*.^11^, and Hannisdal *et al*.^12^). However, the challenge confronting realization of this concept for *in situ* downhole application remains in the limitations of the state of the art in infrared detector technologies. Fundamentally these limitations derive from poor detectivities at the modulation frequencies and elevated temperatures that would be required for logging environments. Conventional FTIR systems that offer the needed detectivity performance at sample rates rapid enough to be of interest for real-time analysis are based upon superconductivity and/or actively cooled detector technologies that typically operate over a narrow bandwidth of the IR spectrum. This renders these systems impractical for real time *in situ* downhole logging applications. To enable an FTIR concept for *in situ* downhole logging, a completely different type of detector technology is needed that, firstly, requires no active cooling in order to achieve the detectivity which would enable real time analysis, and secondly, operates over the full IR range of interest for reservoir fluid chemical analyses. Realization of such a technology has been outside the capabilities of the current state of the art for uncooled detector technologies, but may lie within the framework of solutions achievable using the science of electromagnetic metamaterials.

## Inverted Rhodonea Metamaterial

The current state of the art in uncooled infrared detector technologies provide detectivity and frame rate parameters that continue to be insufficient for practical realization of an infrared detector technology adaptable to downhole logging applications. We have identified a surface geometry based upon mathematical transformation of a canonical conformal mapping that results in a metasurface pattern of subwavelength resonators leading to a detector design exhibiting nearly perfect infrared absorption of 90–100% over a broad infrared bandwidth in the wavenumber range 4500–1700 cm^−1^. The metasurface geometry is integrated with a single Vanadium Dioxide (VO_2_) thermometric substrate in the uncooled microbolometer design with predicted maximum detectivity D* of $1.5\times {10}^{9}\,\text{cm}\sqrt{\text{Hz}}/\text{W}$, and a noise equivalent difference temperature NEDT of 70 mK at a modulation frequency of 60 Hz. This infrared bandwidth is of particular interest for chemical analysis of downhole fluids in hydrocarbon production as many of the primary chemical functional groups of hydrocarbons and gas saturated borehole fluids exhibit electromagnetic resonance signatures in this bandwidth. The metasurface forms the basis of an uncooled microbolometer design that is envisaged for conceptual FTIR detectors applicable to downhole reservoir fluid chemical analysis at sampling frequencies amenable to real time evaluation. The infrared metamaterial detector we describe here is a self-supporting, low mass, micromachined bridge structure that exploits the unusual absorbance characteristics of the metasurface to achieve broadband high detectivity while eliminating the thermal mass associated with conventional absorbing and supporting material layers. The metasurface geometry is imprinted onto the surface of a single thermometric layer of Vanadium Dioxide (VO_2_) acting as the detector thermometer. Due to the broadband high absorptivity of the ultrathin (*λ*/165) metasurface the intrinsic thermal mass of the micobolometer is reduced such that conventional support layers can be eliminated entirely, thereby reducing thermal mass to improve sensor detectivity and total noise level of the detector.

The metasurface geometry is based upon geometric inversion of the canonical Rhodonea conformal mapping contours. Consider the conformal mapping from Cartesian coordinate space to a new virtual domain described by the relations (Field Theory handbook^13^):

1 $$x=\frac{1}{\rho }\sqrt{\rho +u}$$

2 $$y=\frac{1}{\rho }\sqrt{\rho -u}$$

3 $$\rho =\sqrt{{u}^{2}+{v}^{2}}$$

We then consider geometric inversion of the conformal contours using the relations:

4 $$\hat{x}=\frac{1}{\rho }\sqrt{\rho +u}-\sqrt{\frac{8}{u}}$$

5 $$\hat{y}=\frac{1}{\rho }\sqrt{\rho -u}$$

**Figure 1:**
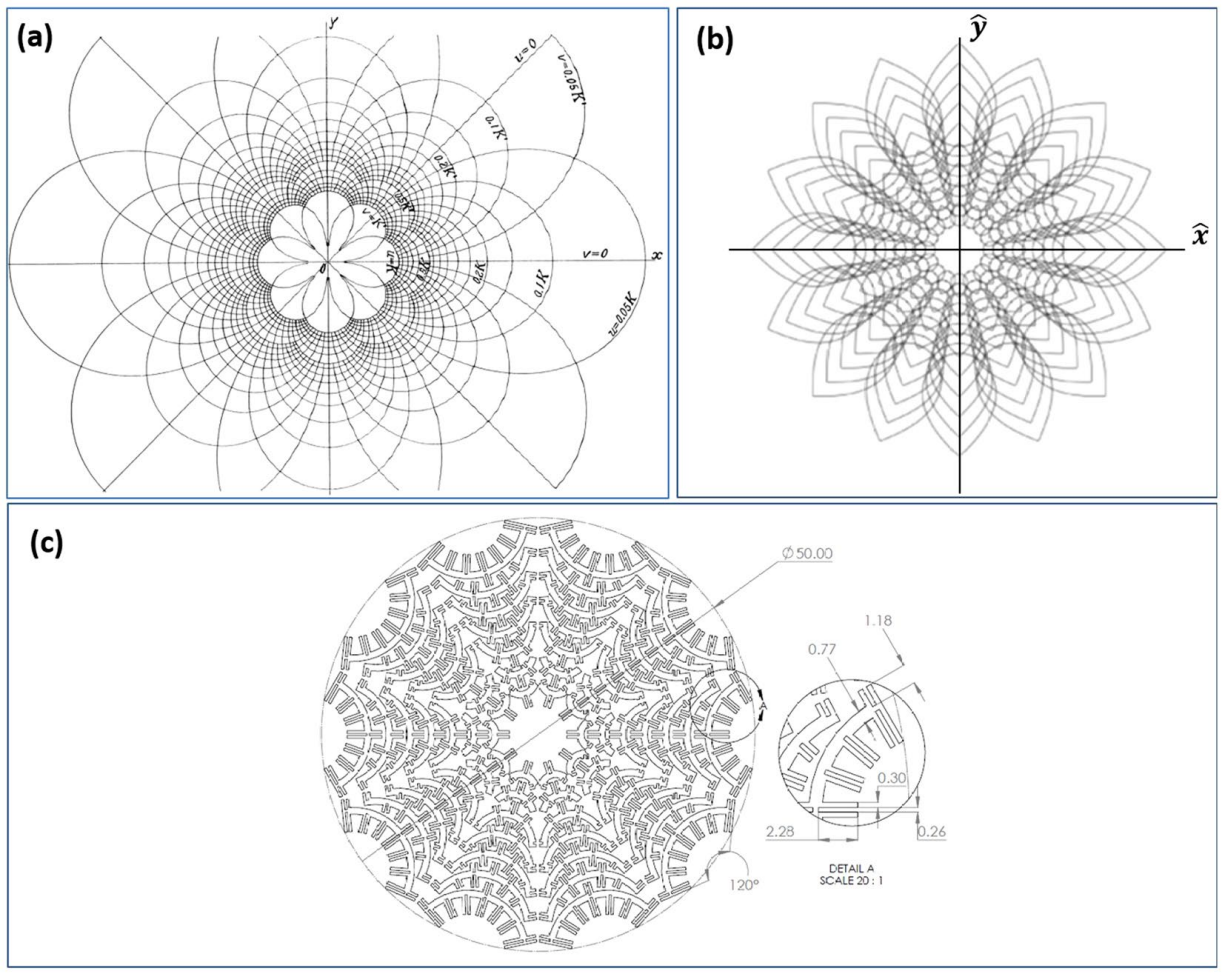
Metasurface geometry formation, (**a**) base Rhodonea conformal mapping contours^13^, (**b**) geometric inversion of base conformal map, (**c**) final metasurface geometry formed along inversion contours.

This geometry transformation gives a new set of non-conformal contours that in effect ‘invert’ the original Rhodonea geometry from inside-out. A graphical illustration of the original conformal contours transformed to the inverted contours and eventually to a metasurface geometry is shown in Fig. 1. A detail of a subcell of the metasurface geometry is depicted in the lower righthand of Fig. 1.

## Effective Electromagnetic Properties

Methods for the determination of effective electromagnetic properties of metamaterials have been investigated extensively^14–18^, and the conventional approaches are based upon retrieval of a set of scattering parameters from a unit cell by plane wave simulations or tests. The origin of the *S*-parameters concept derives from transmission-line theory that is based upon transmitted and reflected voltage waves. For high-frequency problems, such as of interest here, voltage is not a well-defined variable which makes it necessary to define the *S*-parameters in terms of the electric field. An assumption in the formulation of the *S*-parameters retrieval method is that all ports are assumed to be connected to matched loads or excitations. In other words, it is assumed that there is no reflection directly back onto any port from the far field. Consequently in our simulations both input Port 1 and listening Port 2 are bounded by 3D perfectly matched layers (PML). For the general 2 port scattering simulation, the **S** matrix is defined as:

6 $${\bf{S}}=[\begin{array}{cc}{S}_{11} & {S}_{12}\\ {S}_{21} & {S}_{22}\end{array}]$$

in which *S*_11_ is the voltage reflection coefficient at Port 1, *S*_21_ is the voltage transmission coefficient due to wave propagation from Port 1 to Port 2, *S*_12_ is the voltage transmission coefficient due to wave propagation from Port 2 to Port 1, and *S*_22_ is the voltage reflection coefficient at Port 2. As the metasurface simulations do not include either substrate or reflecting PEC ground-plane, the complete matrix of scattering parameters are retrieved from a single forward propagation simulation from Port 1 to Port 2 that retrieves the parameters *S*_11_, *S*_21_. The time average power reflection/transmission coefficients are obtained as $|{S}_{ij}{|}^{2}$.

**Figure 2:**
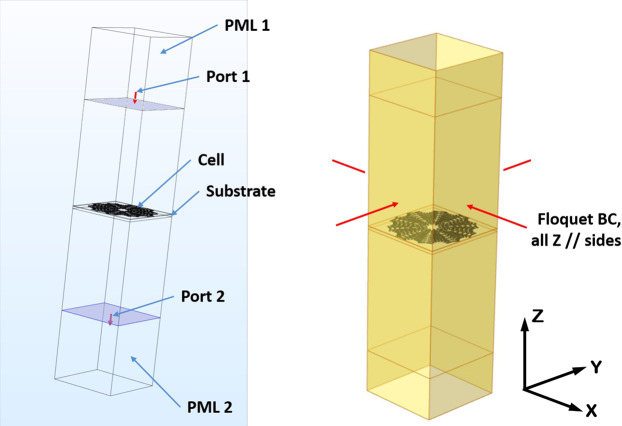
Scattering parameters retrieval finite element model.

In order to determine the absorption characteristics of the metasurface design, and correspondingly the associated detector performance characteristics, firstly a series of electromagnetic scattering parameter simulations was conducted to determine the effective material properties using the commercially available Comsol^®^ MultiPhysics 5.4 finite element analysis software package. The scattering parameters simulation model utilized in the simulations is described in Fig. 2. The simulations were conducted in the form of a 3D periodic waveguide scattering parameter (*S*-parameter) retrieval analysis wherein the metasurface is modeled as a Gold material pattern with a surface transition boundary condition of 27 nm thickness having the geometry described in Fig. 1 with a 50 *μ*m circumscribed diameter. The metasurface Gold material is modeled with an electrical permittivity that is frequency dependent following a Drude model of the form:

7 $${\varepsilon }_{gold}(\omega )=\left[1-\frac{{\omega }_{p}^{2}}{{\omega }^{2}+{\gamma }^{2}}\right]+j\left[\frac{{\omega }_{p}^{2}\gamma }{{\omega }^{3}+\omega \,{\gamma }^{2}}\right]$$

where ${\omega }_{p}=2.164\times {10}^{15}$ Hz is the plasma frequency and $\gamma =16.68\times {10}^{12}$ Hz is the electromagnetic damping frequency. The metasurface mates on the upper surface of a 3D thermometric layer of Vanadium Dioxide (VO_2_) with square area circumscribing the metasurface envelope and having 200 nm thickness. The VO_2_ material was modeled with constant properties of relative permittivity ${\varepsilon }_{r}=10$, relative permeability ${\mu }_{r}=1$, and electrical conductivity $\sigma =50$ S/m. The cross-section of the waveguide coincides with the thermometric layer dimensions. The remainder of the waveguide has the properties of free space (vacuum). The waveguide is excited with an incident transverse magnetic (TM) wave from Port 1 according to:

8 $${\bf{H}}={H}_{0}[0\,{H}_{y}\,0]$$

9 $${H}_{y}={e}^{jkx}\,{e}^{jky}$$

where $k=\omega$/*c* is the wavenumber in free space at the electromagnetic frequency $\omega$, and c is the speed of light in vacuum. A second Port 2 is located symmetrically about the substrate surface from Port 1. The waveguide boundaries perpendicular to the $x$ and $y$ coordinate directions have Floquet periodic boundary conditions that account for the plane wave interaction with periodic structure.

**Figure 3:**
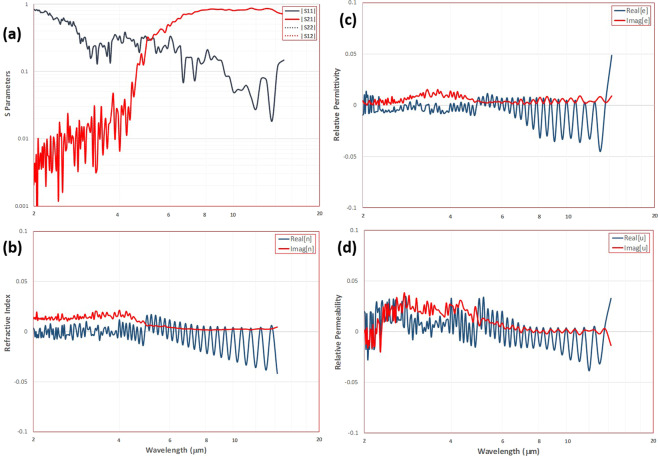
Metasurface simulations. (**a**) Scattering parameters retrieval. (**b**) Effective refractive index spectra. Dark blue line is real part and dark red line is imaginary part of index. (**c**) Electrical permittivity, dark blue line is real part and dark red line is imaginary part of $\varepsilon$. (**d**) Magnetic permeability, dark blue line is real part and dark red line is imaginary part of *μ*.

The retrieved scattering parameters are shown in the spectrum plot of Fig. 3a. For the free metasurface (i.e., no substrate or reflector) simulations, the scattering parameters for forward/backward propagation are identical owing to the absence of a substrate. The effective properties calculations were based upon the retrieval procedure described in Smith *et al*.^19^ with a minor change in the selection of the phase complex root. Markel^20^ makes the case that material passivity fully constrains the imaginary part of permittivity to be positive, but does not preclude negativity in the imaginary part of permeability. As well, Arslanagic *et al*.^21^ show that the parameters, relative impedance and refractive index, are not independent parameters and as such the selection of one root fixes the value of the other. The retrieval process then need only ensure that consistent use be maintained throughout the properties calculations. This is reasonable considering that these parameters are not fundamental quantities in Maxwell’s equations but derived values. Thus, the minor modification we use here is to select the complex phase root corresponding to positivity in the imaginary part of effective refractive index, also fixing the dependent value of relative impedance. Two roots are then found for the fundamental quantities of permittivity and permeability. Passivity constrains the selection to the appropriate root pair maintaining positivity in the imaginary part of the effective permittivity. Therefore the only ambiguity in the retrieval process is branching in the real part of the phase parameter. The retrieval analysis is generated from a sufficiently low frequency to ensure that the retrieval slab thickness is thin compared to the incident wavelength. In this manner, the initial branch number in the analysis is ensured to be $m=0$ and any branching can be observed with increasing frequency by comparison with multiple branch number spectra. In this analysis no branching from $m=0$ was found over the frequency range considered for this inverted mapping geometry.

The resulting refractive index spectrum is shown in Fig. 3b and shows both real and imaginary components that vary closely about zero over the wavelength range of interest (2–15 *μ*m). The corresponding electrical permittivity and magnetic permeability spectra are shown in Fig. 3c,d. Similarly the effective electromagnetic properties have both real and imaginary parts closely to zero over the range of interest. For the free metasurface the absorption spectrum can be calculated using:

10 $$A=1-[|{S}_{11}{|}^{2}+|{S}_{21}{|}^{2}]$$

**Figure 4:**
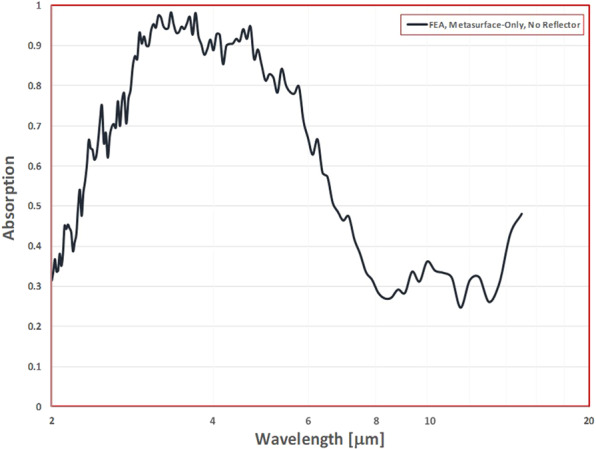
Absorption spectrum, free metasurface (no substrate/reflector).

The resulting absorption spectrum in Fig. 4 for the free metasurface indicates a bandwidth of high absorptivity above 90% in the range between 2.8–4.8 *μ*m. In this bandwidth the inverted Rhodonea metasurface is ultrathin at a small fraction *λ*/165 of the center frequency incident wavelength of 4.5 *μ*m. The imaginary components of the effective parameters spectra shown in Fig. 3 indicate that the metasurface is a very low loss effective medium. Conventional understanding is that materials with intrinsically high loss are necessary to develop high absorptivity in thin layers. Jin *et al*.^22^ showed that theoretically perfect absorption could be achieved at a critical angle of incidence in ultrathin metamaterial structures coupled to a totally reflective substrate when the real parts of the permittivity and permability are zero and the magnitudes of the imaginary parts are arbitraily small. Their analysis indicated that as the imaginary components of the effective electromagnetic properties of the metamaterial approached zero the thickness of the metamaterial was also required to approach zero in order to achieve perfect absorption at the critical angle of incidence. Zhong *et al*.^23^ showed that the critical incidence angle perfect absorption is due to coherent cancellation, that is, the interference of out-of-phase reflected waves between the metamaterial layer and the perfectly reflecting substrate. Feng *et al*.^24^ described theoretically how perfect absorption can occur in an epsilon-near-zero ultrathin metamaterial on a metal substrate. They attribute the perfect absorption phenomena to above-light-line surface plasmon polaritons that arise at the interface between the metasurface and the metal layer, inducing fast-wave non-radiative modes in the bilayer. We hypothesize here that the fundamental absorption phenomena for the inverted Rhodonea metasurface here is associated with surface plasmon resonances, but do not consider an in depth investigation with this work.

**Figure 5:**
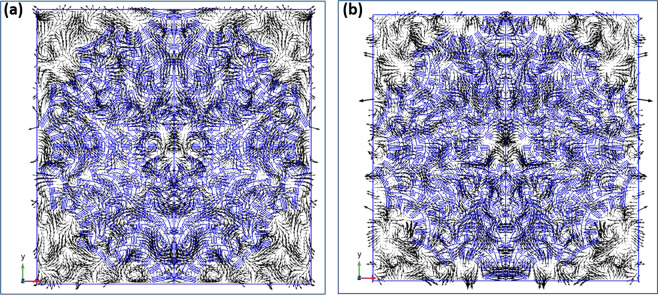
Electromagnetic contour lines on the metasurface plane at 81 THz (3.7 *μ*m), (**a**) Electric field, (**b**) Magnetic field.

The distribution of the electromagnetic fields at a representative perfect absorption frequency of 81 THz (3.7 *μ*m) is illustrated in the plots of Fig. 5. The contours of the electric field lines are shown in Fig. 5(a) and the magnetic field lines are shown in Fig. 5(b). The interaction of the incident plane wave radiation with the inverted Rhodonea geometry pattern is characterized by a series of local vorticity regions in the field lines of both the electric and magnetic fields, being distributed throughout the metasurface envelope footprint and into the free space corners between patterns. The power dissipation mechanism is comprised of resistive losses in the metal metasurface with no magnetic losses.

**Figure 6:**
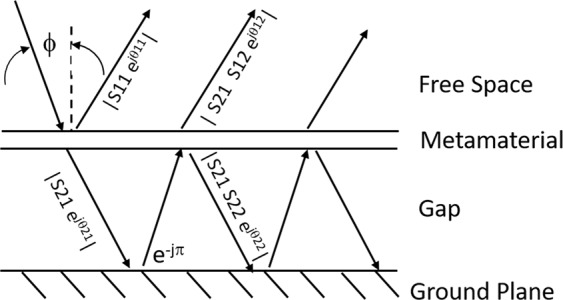
Multiple reflection model for metamaterial/spaced-reflector interference analysis.

The introduction of a spaced reflector behind the Gold metasurface may enhance this absorption spectrum, with the proper spacing. Chen^25^ showed that the mechanism for absorption from the introduction of a spaced reflector behind a metamaterial slab is due almost entirely to reflection interference. He presented an equation to describe the resulting absorption in which the metamaterial absorber is coupled to the spaced reflector only by multiple reflections with no near-field interactions. We use that approach to evaluate analytically a range of reflector spacing to determine the optimum value that maximizes absorption magnitude and bandwidth. To illustrate the analysis consider Fig. 6 showing the ray propagation of an incidence on a metamaterial slab with a perfectly reflecting ground plane spaced distance ${d}_{g}$ behind. For incident wave with electric field **E**_*i*_, a typical ray encounters the metamaterial and is partially reflected and partially transmitted. The transmitted portion reflects from the perfectly conducting ground plane and again encounters the metamaterial having acquired a propagation phase change of ${\phi }_{0}=2\beta +\pi$, where $\beta ={k}_{0}{d}_{g}$ for normally incident ray, and the additional $\pi$ phase change is a result of the reversal in direction at the ground plane. This internal reflection then similarly is partially reflected back to the ground plane and partially transmitted back into the incidence medium. The process repeats for an indefinite number of cycles. The total portion of the incident electric field ray that is reflected back into the incidence medium is then the superposition of the initial incidence reflection and all the re-transmitted portions of the internally reflecting rays. The resulting modified reflectivity parameter ${\tilde{S}}_{11}$ is then given by:

11 $$\begin{array}{rcl}{\tilde{S}}_{11} & = & \frac{{E}_{reflect}}{{E}_{i}}\\ {\tilde{S}}_{11} & = & {S}_{11}-\left[\frac{{S}_{21}{S}_{12}{e}^{j2\beta }}{1+{S}_{22}{e}^{j2\beta }}\right]\end{array}$$

**Figure 7:**
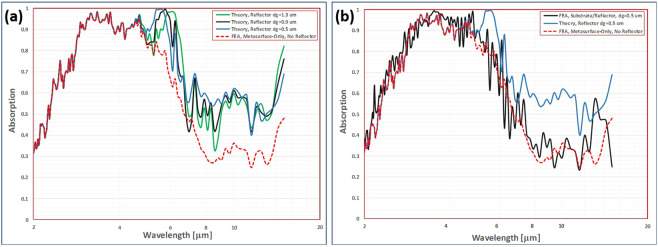
Reflector spacing trade study. (**a**) Different absorption spectra with changes in reflector spacing *d*_*g*_ using the analytical interference calculation in Eq. (11). (**b**) Absorption spectra: Solid dark blue line is detailed FEA using reflector with spacing *d*_*g*_ = 0.5 *μ*m, Dashed red line is analytical calculation based on Eq. (11) with reflection interference mechanism only, light blue line is detailed FEA for metamaterial cell only with no reflector.

An analysis was conducted using the prediction for modified reflectivity in Eq. (11) to calculate changes in absorption spectra as a function of reflector spacing ${d}_{g}$. The criterion for selection of the optimum reflector spacing was maximum of the integral of absorption spectra between 2–7 *μ*m. The theoretical analyses indicate that some enhancement can be accomplished with the incorporation of a spaced reflector, primarily in the wavelength range above 5 *μ*m with negligible effect on absorption below 5 *μ*m. A maximum in absorption spectra integral was found with a reflector spacing ${d}_{g}=0.5\,\mu \text{m}$ in the range investigated $0.1\,\mu \text{m} < {d}_{g} < 2.0\,\mu \text{m}$. A comparison of the absorption spectra without a reflector and with various reflector spacing including the optimum spacing ${d}_{g}=0.5\,\mu \text{m}$ is shown in Fig. 7a. The theoretical analysis indicates the 80–100% absorption bandwidth should be broadened using an optimized reflector spacing. A subsequent finite element analysis incorporating a PEC reflector spaced at a gap distance of ${d}_{g}=0.5$ *μ*m is shown in the comparison of Fig. 7b. The detailed FEA simulations indicate that incorporation of the reflector has much less of a broadening effect on the absorption bandwidth than predicted by the analytical results using Eq. (11). In fact, the resulting absorption bandwidth after incorporation of the reflector is only slightly improved over the free metasurface-only spectrum.

**Figure 8:**
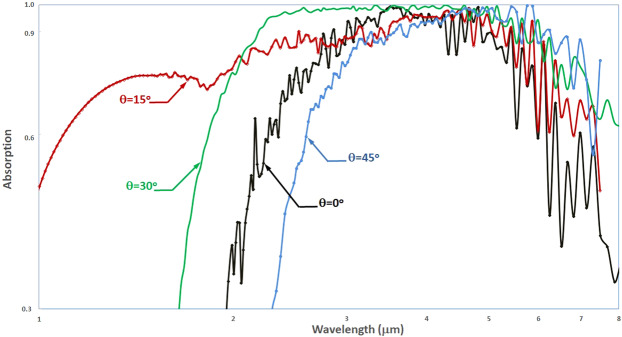
Metasurface detector absorption spectra for varying incidence angle *θ* relative to surface normal. Metasurface is Gold at 27 nm thickness, thermometric substrate is VO_2_ at 200 nm thickness, with a PEC reflector spaced 500 nm from substrate.

Additional analyses considering the effects of deviation of the incident wave from the surface normal were conducted to understand the sensitivity of the metasurface absorption to detector mounting orientation relative to the field of view. The absorption spectra for varying incidence angle between 0°–45° from normal are shown in Fig. 8. The absorption spectra comparison reveals a significant enhancement of the bandwidth as the incidence angle approaches 30° from normal, particularly over the short wavelength range down to 1 *μ*m. The broadest 90–100% absorption bandwidth is seen at a 30° incidence angle, ranging between 2.2–6.0 *μ*m, with a 170% FWHM centered about 15.8 *μ*m.

**Figure 9:**
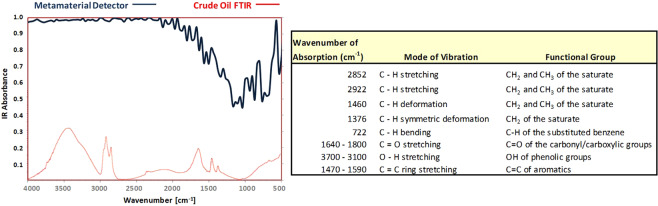
Metasurface detector characteristics, (L) absorption spectra overlay for inverted metasurface detector and FTIR for representative crude oil sample from Samanta^26^, (R) functional groups in the IR spectrum of the crude oil from Samanta^26^.

The IR absorption spectrum for the metasurface detector at 30° incidence is shown in the left-hand side of Fig. 9 superimposed with the FTIR absorption spectrum for a crude oil from Samanta *et al*.^26^ for comparison. The crude oil measured by Samanta originated from the Ahmedabad oil field (India) and has an API gravity of 38.86° and viscosity of 119 cPs at 30 °C. A description of the spectral functional groups modes of vibration for the crude oil is summarized in the right-hand side of Fig. 9, also from Samanta *et al*.^26^. The metasurface detector absorption is more than 98% over the higher wavenumber range from 4000 cm^−1^ to 2000 cm^−1^, indicating potentially good discrimination of the CH2, CH3, and OH (phenolic) functional groups of the crude oil absorbance peaks. The detector absorption decreases to 50–70% for the lower wavenumber range 2000 cm^−1^ to 500 cm^−1^ which includes the C=O (carbonyl), C=C (aromatics), C-H (substituted benzene), and CH_2_ & CH_3_ saturate functional groups. The metasurface detector exhibits a maximum absorption of 99.94% at 4.3 *μ*m and an absorption bandwidth of 170% full-width half-maximum (FWHM) on 15.8 *μ*m center wavelength.

## Metasurface Detector Performance

**Figure 10:**
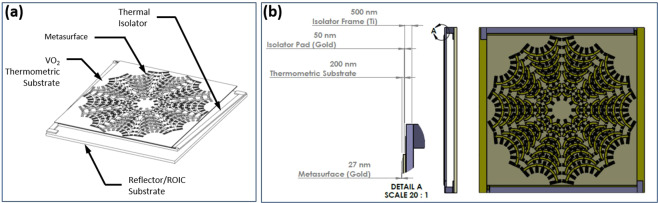
Uncooled microbolometer design concept, (**a**) Annotated isometric, (**b**) Detailed elevations.

Conventional microbolometer detector devices are usually designed having a micromachined bridge structure suspended over a readout integrated circuit (ROIC) substrate in which one of the materials comprising the laminated bridge structure is a thermometric layer that experiences a change in electrical resistance with temperature change, while other layers function to absorb the infrared energy and support the overall bridge structure. The similar type bolometer concept considered here for the metasurface detector design is illustrated in Fig. 10.

The change in resistance of the thermometric layer is the response detected by the ROIC in the form of a change in voltage drop across the contacts of the bridge under constant bias current. The bridge structure is suspended over the substrate with an air gap in order to minimize the thermal conduction path to heat generated in the absorbing layer, allowing the ROIC to compound the effects of incident radiation and enhance the electrical signal created in response to changing field thermography. A reduced thermal conduction path, though, must be balanced against increasing the thermal time constant and reducing the responsiveness to changing incident radiation. The bridge mass can be reduced in order to improve the response time, but conventionally results in a loss of infrared absorptivity and an increase in voltage noise level on the detector. More rapid frame rates limit responsivity and detectivity while increased temperatures contribute to noise levels, which have historically combined to limit the operational regime of conventional uncooled microbolometer technologies to low frequency room temperature applications. High performance applications involving near-background radiation limited performance at rapid frame rates have been limited in practice to systems with active cooling. In the next section we investigate the predicted detector performance characteristics from integration of the inverted Rhodonea metasurface into a conventional uncooled microbolometer architecture.

## Detector Figures of Merit

The comparative performance of detectors has conventionally been characterized using three parameters as figures of merit. These are voltage responsivity (*R*_*v*_), signal to noise detectivity (D*), and total voltage noise level usually given in terms of a noise equivalent difference temperature (NEDT). The voltage responsivity *R*_*v*_ is a function of the output voltage signal and the temperature responsivity with changes in incident electromagnetic flux on the detector, and is given by the relation^27^.

12 $${R}_{v}={I}_{b}\,R\,\beta \,{R}_{T}={I}_{b}\,R\,\beta \frac{\Delta \bar{T}}{\Delta {\bar{\phi }}_{0}}$$

where:

*I*_b_: bias current (A),

*R*: bolometer electrical resistance (Ω),

*β*: thermometric layer temperature coefficient of resistance (TCR, 1/K),

*R*_T_: temperature responsivity of the detector (K/W),

Δ$\bar{T}$: complex variation in temperature of the detector (K),

Δ${\bar{\phi }}_{0}$: complex variation in incident radiation flux (W).

The temperature change of the detector is a result of the combined effects from thermal conduction through the contact leads, and radiation and convection from the detector surfaces. Therefore, we can write for the equation of heat balance in the detector:

13 $${M}_{th}\frac{\partial \Delta \bar{T}}{\partial t}+{G}_{th}\Delta \bar{T}=\eta \Delta {\bar{\varPhi }}_{0}$$

where *M*_*th*_ is the cumulative heat capacity of the detector, *G*_*th*_ is the cumulative thermal conductance of the detector, $\eta$ is the fraction of incident electromagnetic flux absorbed by the detector, and Δ${\bar{\varPhi }}_{0}$ is the variation in the incident electromagnetic flux on the detector. The cumulative heat capacity, in the general laminate case, is given by:

14 $${M}_{th}={A}_{d}\,\sum _{N}\,{m}_{n}{\rho }_{n}{t}_{n}$$

where ${m}_{n}$ is the heat capacity, ${\rho }_{n}$ is the mass density, ${t}_{n}$ is the thickness of each layer 1:*N*, and ${A}_{d}$ is the detector area of radiation incidence. The convective losses can be ignored when the detector is mounted in a hard-vacuum cavity, and the remaining thermal conductance is highly dominated by the conduction through the contact lead structures. Therefore, the cumulative thermal conductance ${G}_{th}$ can be given by:

15 $${G}_{th}=\sum _{M}\,{\kappa }_{m}\frac{{A}_{m}}{Lm}$$

where ${\kappa }_{m}$ is the thermal conductivity, ${A}_{m}$ is the cross-sectional area, ${L}_{m}$ is the length of each contact lead 1:*M*, the number of contact leads between the bridge structure and the substrate. Then, for voltage responsivity ${R}_{v}$ (V/W) we have:

16 $${R}_{v}=\frac{{I}_{b}\,R\,\beta \,\eta }{{G}_{th}\sqrt{1+{\omega }^{2}{\tau }_{th}^{2}}}$$

where ${\tau }_{th}=\frac{{M}_{th}}{{G}_{th}}$ is the thermal time constant of the detector and *β* is the temperature coefficient of resistance for the thermometric substrate. It was found by Jones^28^ that for many detectors the signal to noise ratio is a function of the square root of the product of detector area and the modulation bandwidth, and a normalized detectivity can then be calculated as:

17 $${D}^{\ast }=\frac{{R}_{v}}{\Delta {V}_{n}}\sqrt{\Delta f{A}_{d}}$$

where Δ*f* is the electrical amplifier bandwidth, and Δ*V*_*n*_ is the total voltage noise of the detector. The total voltage noise includes Johnson, or thermal, noise as well as $\frac{1}{f}$ noise seen at low frequencies. The Johnson noise is given by:

18 $$\frac{\Delta {V}_{n,j}^{2}}{\Delta f}=4kT\,R$$

where $k=1.38\times {10}^{-23}$ n-m/K is Boltzmann’s constant, and $T$ is the absolute temperature (K) of the bridge structure. The $\frac{1}{f}$ noise can be estimated using the Hooge^29^ relation for a homogenous semiconductor film:

19 $${\left[\frac{\Delta R}{R}\right]}^{2}={\alpha }_{H}\left[\frac{\Delta f}{N\,f}\right]$$

where ${\alpha }_{H}=0.002$ is the Hooge coefficient for homogenous semiconductor films, $f$ is the modulation frequency, Δ*f* is the modulation bandwidth, and $N$ is the number of free carriers (electrons) in the sample. We can rewrite the Hooge equation Eq. (19) in the form:

20 $$\frac{\Delta {V}_{n,H}^{2}}{\Delta f}={\alpha }_{H}\left[\frac{{I}_{b}^{2}{R}^{2}}{Nf}\right]$$

and obtain as an estimate for the total voltage noise in the detector:

21 $$\frac{\Delta {V}_{n}^{2}}{\Delta f}=4kTR+{\alpha }_{H}\left[\frac{{I}_{b}^{2}{R}^{2}}{Nf}\right]$$

The detector signal to noise detectivity D* is then written more explicitly as:

22 $${D}^{\ast }={R}_{v}\sqrt{\frac{{A}_{d}}{4kTR+{\alpha }_{H}\left[\frac{{I}_{b}^{2}{R}^{2}}{Nf}\right]}}$$

Noise equivalent difference temperature (NEDT) denotes the temperature change of a detector due to incident radiation that corresponds to an output signal equal to the rms total noise level (a signal-to-noise ratio of 1). This is a fundamental parameter of the detector performance and represents the minimum temperature difference that can be discerned above the background noise, and is given by the relation:

23 $$\text{NEDT}=\Delta {V}_{n}\frac{\Delta T}{\Delta {V}_{s}}=\Delta {V}_{n}\frac{{R}_{T}}{{R}_{v}}$$

where Δ*V*_*s*_ is the voltage change for a temperature change of Δ*T* on the detector, and Δ*V*_*n*_ is the rms total noise voltage level.

## Detector Performance Predictions

The metasurface detector design is based upon integration with a single thermometric layer of VO_2_ having no supporting layers. This is possible due to the low mass loading from the ultrathin Gold metasurface layer. Specifically, the metasurface geometry has a 35% fill factor within a 50 *μ*m diameter. For a 200 nm VO_2_ substrate thickness and 27 nm Gold metasurface thickness, the resultant mass loading develops a maximum bending stress in the substrate of 354 Pa/g. The tensile strength of the VO_2_ substrate is ${\sigma }_{ult}=172$ MPa giving an ultimate shock capability of $486\times {10}^{3}$ g’s, much greater than required to sustain the expected worst case shock loads that could be experienced downhole (<1000 g’s). As a result, confinement to a single thermometric layer is acceptable with this ultrathin metasurface for the expected downhole vibration and shock environments.

**Figure 11:**
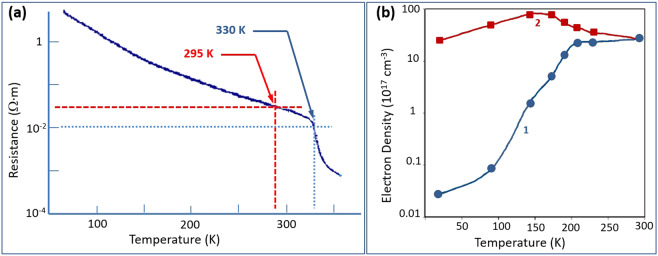
VO_2_ properties: (**a**) Temperature dependence of electrical resistivity for undoped VO_2_ (from Pergament^30^). (**b**) Theoretical calculations for the temperature dependence of electron density in VO_2_ film, for (1) assuming constant electron mobility, and (2) assuming temperature dependent electron mobility (from Pergament^31^).

**Figure 12:**
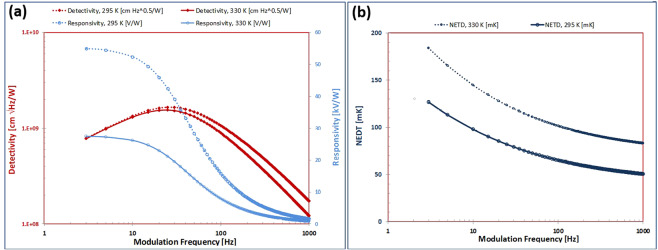
Metasurface detector performance predictions as a function of modulation frequency and temperature, at 90% absorption, (**a**) voltage responsivity ${R}_{v}$ and normalized detectivity D*, and (**b**) noise equivalent difference temperature NEDT.

The normalized detectivity as given by Eq. (22) is dependent upon the electrical resistivity of the substrate material, VO_2_ film, while the noise equivalent difference temperature (NEDT) is dependent upon its specific carrier density. The trends of the electrical resistivity properties of VO_2_ films with temperature were measured by Pergament *et al*.^30^, and are shown in Fig. 11a, which clearly illustrates the metal-insulator-transition (MIT). For undoped VO_2_ film the data in Fig. 11a indicates a room temperature resistivity of approximately 2 Ω-cm, which decreases to a value of approximately 1 Ω-cm at the initiation of the MIT at around 35 °C. The electron density of VO_2_ has been calculated based upon theoretical considerations by Pergament *et al*.^31^, and shown in Fig. 11b. From the theoretical calculations we see that the predicted estimates for electron density converge for both constant mobility and temperature dependent mobility assumptions at room temperature with a value of approximately 4 × 10^18^/cm^3^. Using these material properties for the VO_2_ thermometric layer, along with Eq. (16) for responsivity ${R}_{v}$, Eq. (17) for normalized detectivity D*, and Eq. (23) for noise equivalent difference temperature NEDT and the Comsol^®^ MultiPhysics simulation results for the metasurface pixel absorptivity of Fig. 9, we can now evaluate predictions for the metasurface detector performance figures of merit. Using the detector figures of merit as a set of discriminators, a series of analytical trade-off studies was conducted to optimize room temperature detector performance for a 60 Hz modulation frequency. The optimized results are summarized in Fig. 12 which shows the results for detector trends in responsivity, detectivity, and NEDT as a function of modulation frequency at temperatures of 295 K and 330 K. The results in Fig. 12 are based upon a uniform IR absorption ratio of 0.9. The detector figures of merit for 0.9 absorption normalization, modulation frequency *f* = 60 Hz and modulation bandwidth Δ*f* = 10 Hz are detectivity D* of $1.35\times {10}^{9}\,\text{cm}\sqrt{\text{Hz}}$/W and $1.18\times {10}^{9}\,\text{cm}\sqrt{\text{Hz}}$/W at 295 K and 330 K, respectively, and NEDT of 70 mK and 108 mK at 295 K and 330 K, respectively. The results are based upon 5 *μ*A bias current which creates a latent temperature rise of 4 K in the microbolometer.

**Figure 13:**
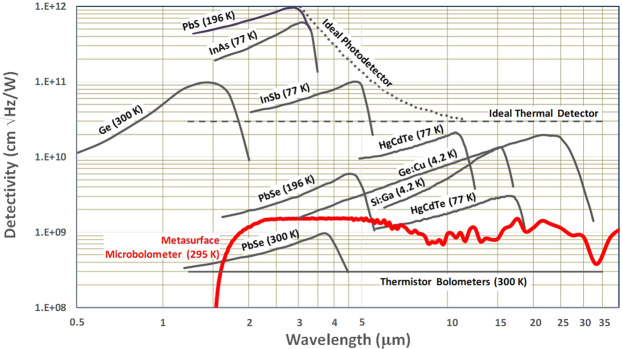
Comparison of spectral detectivity for the metasurface detector [RED], and various commercially available IR detectors (Hamamatsu Corp. (Hamamatsu Corp. Characteristics and use of infrared detectors. Technical Information SD-12, Cat. No. KIRD9001E04)) operated at the noted temperatures. The modulation frequency for all detectors is 1000 Hz, except for the state of the art uncooled thermistor bolometers at 10 Hz and the metasurface detector at 60 Hz.

The direct comparison in Fig. 13 includes the detectivity spectrum of the metasurface detector at 60 Hz modulation frequency superimposed onto the spectra for various commercially available infrared detector technologies operated at the noted temperatures and over the wavelength range from 0.5–40 *μ*m. The superimposed metasurface detector spectrum indicates a maximum detectivity D* of $1.5\times {10}^{9}\,\text{cm}\sqrt{\text{Hz}}$/W, which is near the lower range of performance for the state of the art cryogenically cooled detectors in the comparison. Nonetheless, this would represent a disruptive technology for uncooled microbolometers in consideration of the comparison with such as a photon detection based Ge:Cu cooled to 4.2 K, PbSe cooled to 196 K, and a photovoltaic HgCdTe cooled to 77 K. The details of the metasurface detector design are summarized in Table 1.

**Table 1:** Summary of metamaterial microbolometer design properties.

VO_2_ substrate dimensions	52 × 52 × 0.20 mm^3^
Metasurface envelope	50 × 0.027 *μ*m^3^
Maximum Absorption	99.94%
Ti electrode dimensions (2)	46.0 × 1.0 × 0.25 *μ*m^3^
Resistance, *R*	100 kΩ
Bias Current, *I*_*b*_	5 *μ*A
Temperature Rise, Δ${T}_{{I}_{b}}$	4.0 K
TCR, *β*	0.03 1/K
Thermal Conductance, *G*_*th*_	2.45 × 10^−7^ W/K
Thermal Capacitance, *C*_*th*_	1.34 × 10^−9^ J/K
Thermal Time Constant, *τ*_*th*_	5.3 ms
Maximum Responsivity, *R*_*v*_	24.7 × 10^3^ V/W @ 60 Hz
Maximum Detectivity, D*	1.5 × 10^9^ cm$\sqrt{\text{Hz}}$/W @ 60 Hz

## Conclusion

In this paper we have presented a perfect metasurface absorber (PMA) design formed from a set of inverted conformal contours of the Rhodonea, or more commonly four-leaf roses, conformal mapping. The PMA behaves as a near zero index metamaterial (NZIM) having intrinsic multiple coupled absorption resonances that combine to form broadband infrared absorption characteristics of 100% FWHM bandwidth on 4.5 *μ*m center wavelength. Scattering parameter retrieval simulations indicate the metasurface absorbs between 80–100% of incident infrared radiation in the range 2.8–5.8 *μ*m. An uncooled microbolometer design is described that uses the metasurface geometry on a single Vanadium Dioxide (VO_2_) thermometric substrate leading to an infrared detector with predicted maximum absorption of 99.94% at 4.3 *μ*m and an absorption bandwidth of 170% full-width half-maximum (FWHM) on 15.8 *μ*m center wavelength, coincident with important chemical spectra of downhole hydrocarbons. Figures of merit analyses for the uncooled microbolometer result in predicted maximum detectivity ${\text{D}}^{\ast }=1.5\times {10}^{9}\,\text{cm}\sqrt{\text{Hz}}/\text{W}$ and noise equivalent difference temperature NEDT of 70 mK at a frame rate of 60 Hz and temperature of 295 K. These parameters could make the technology viable for downhole application of *in situ* FTIR spectroscopy.

## Supplementary information

Supplementary Information.

## Data Availability

All data analysed during this study are included in this published article.
