# Supplementary figures and images for: Near Zero Index Perfect Metasurface Absorber using Inverted Conformal Mapping
Source: Sci Rep. 2020 Jun 16;10:9731. doi: 10.1038/s41598-020-66476-x (PMC7297995; doi:10.1038/s41598-020-66476-x)

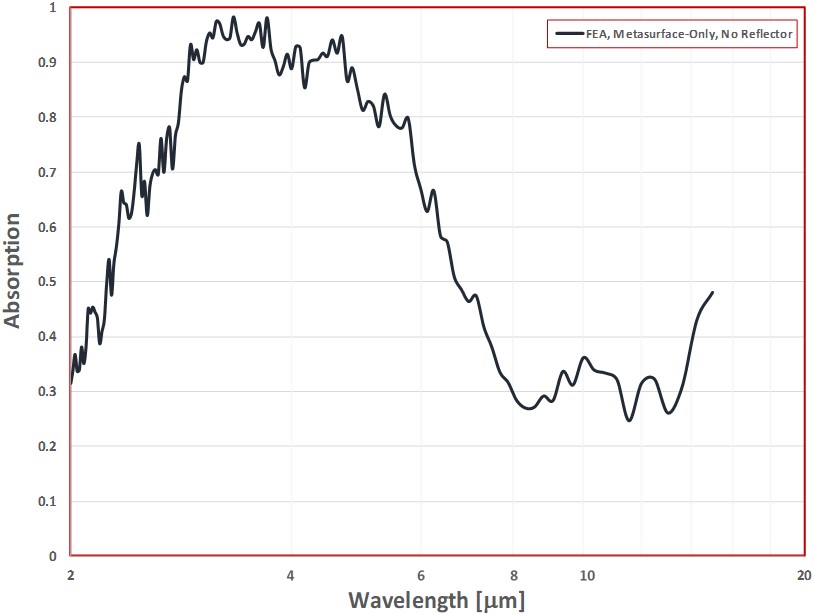

Supplement: Supplementary file 1 — Supplementary Information. [file 41598_2020_66476_MOESM1_ESM.zip › Figures_titled_jpg_3-24-2020/Cell-A_Plot.jpg]

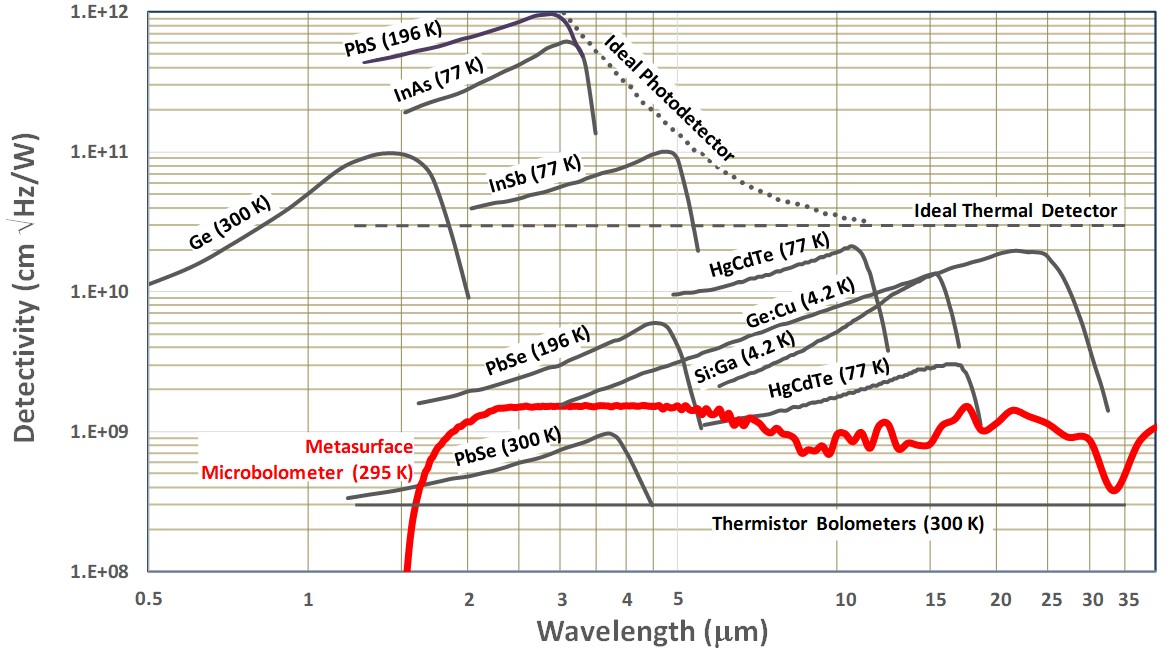

Supplement: Supplementary file 1 — Supplementary Information. [file 41598_2020_66476_MOESM1_ESM.zip › Figures_titled_jpg_3-24-2020/Compare-Detectors_Plot-5.jpg]

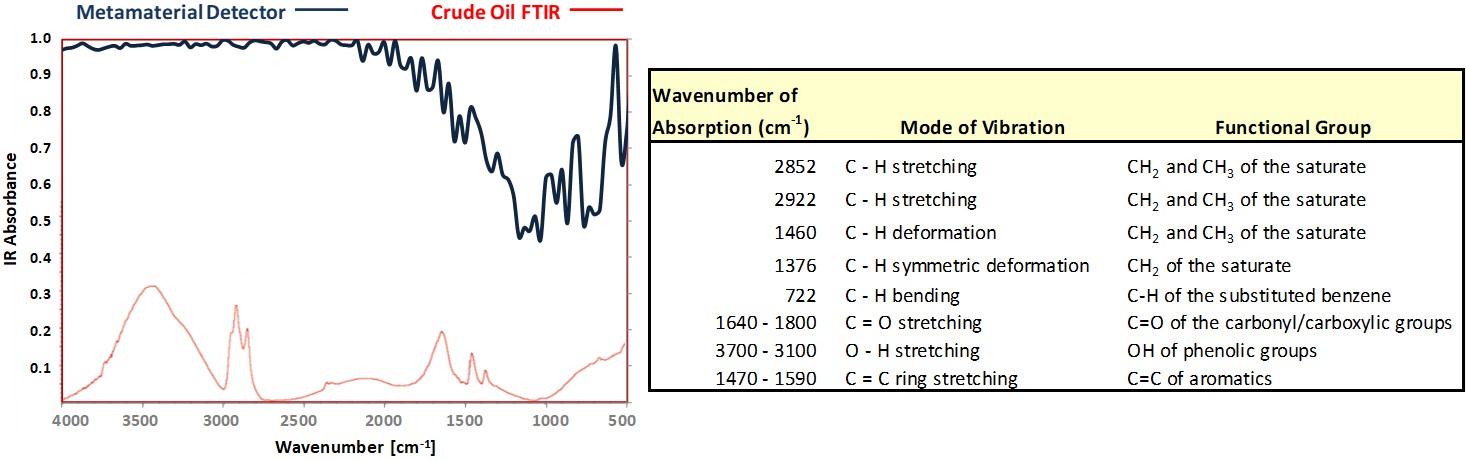

Supplement: Supplementary file 1 — Supplementary Information. [file 41598_2020_66476_MOESM1_ESM.zip › Figures_titled_jpg_3-24-2020/Detector_Crude_Spectra_Overlay-2.jpg]

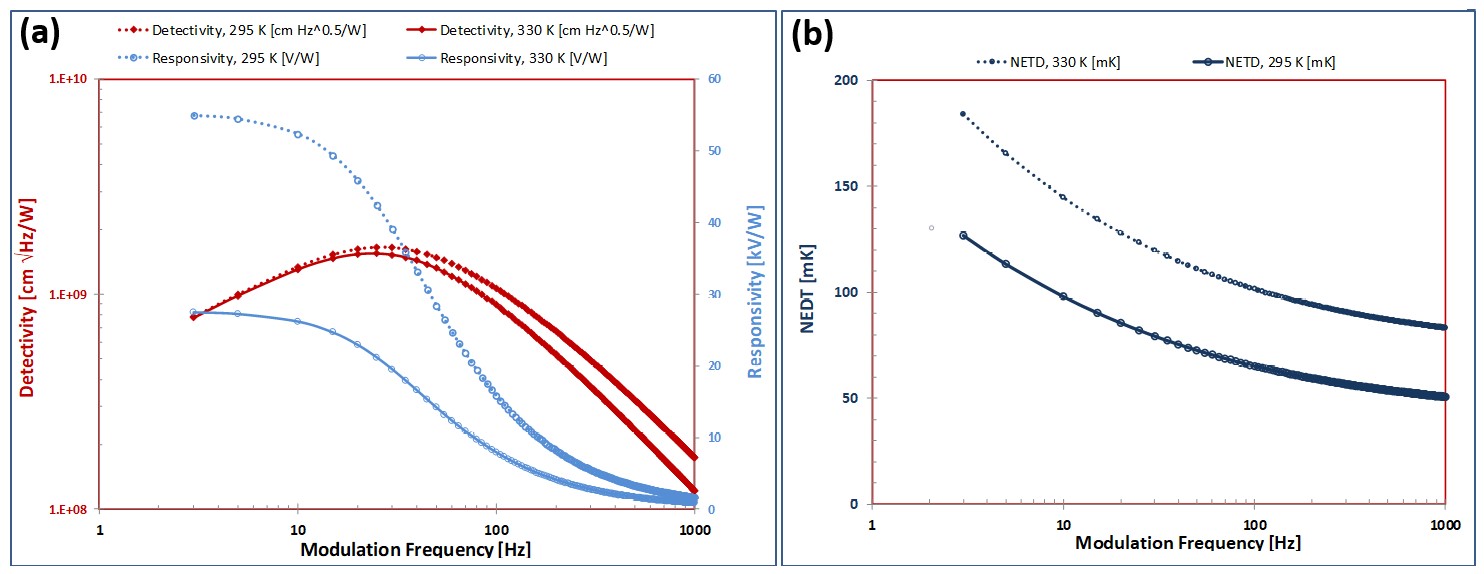

Supplement: Supplementary file 1 — Supplementary Information. [file 41598_2020_66476_MOESM1_ESM.zip › Figures_titled_jpg_3-24-2020/Detector-Response_Plot.jpg]

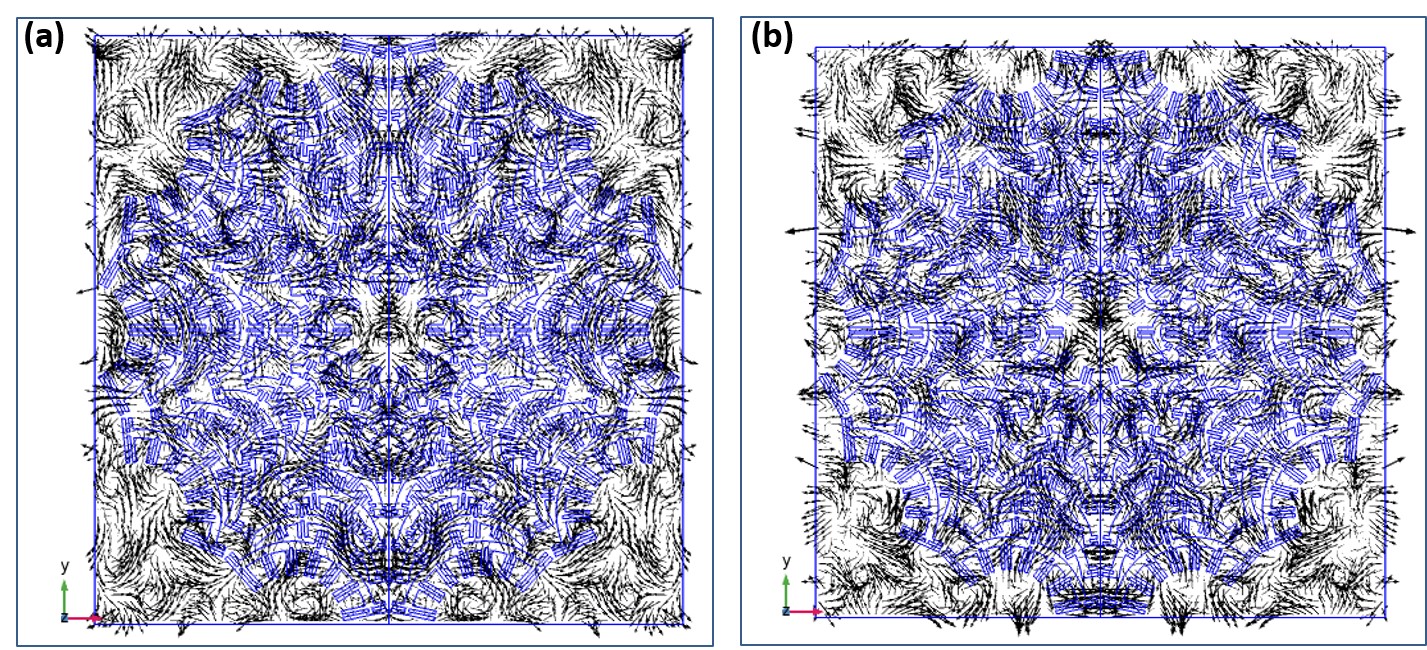

Supplement: Supplementary file 1 — Supplementary Information. [file 41598_2020_66476_MOESM1_ESM.zip › Figures_titled_jpg_3-24-2020/Dual_EM-Fields_Plot.jpg]

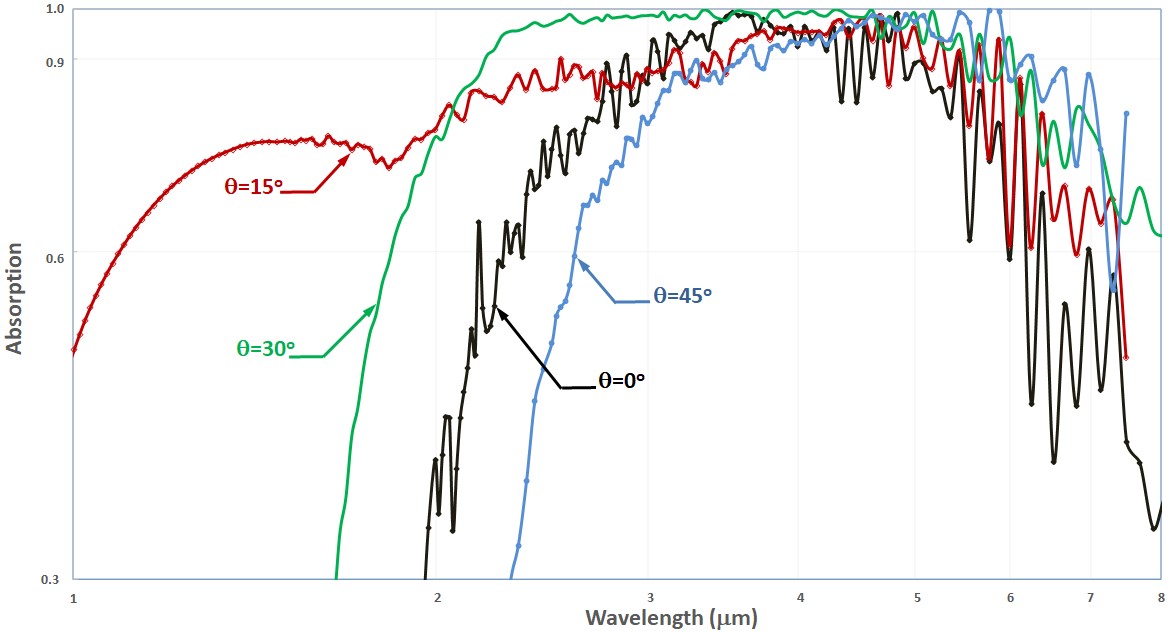

Supplement: Supplementary file 1 — Supplementary Information. [file 41598_2020_66476_MOESM1_ESM.zip › Figures_titled_jpg_3-24-2020/Incidence-Angle_Plot.jpg]

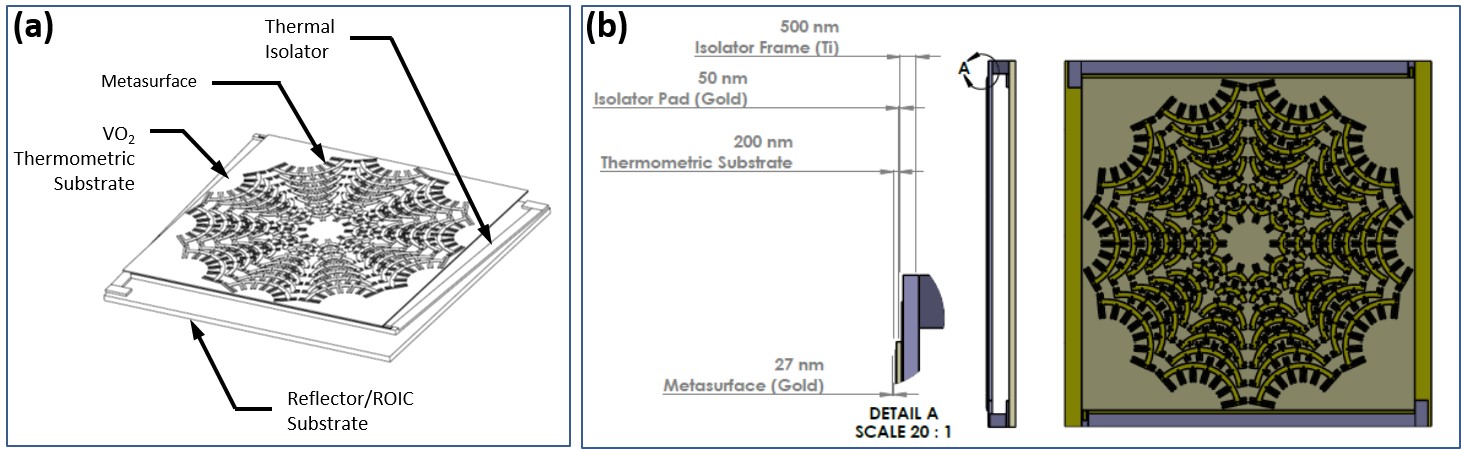

Supplement: Supplementary file 1 — Supplementary Information. [file 41598_2020_66476_MOESM1_ESM.zip › Figures_titled_jpg_3-24-2020/Microbolometer_Assy.jpg]

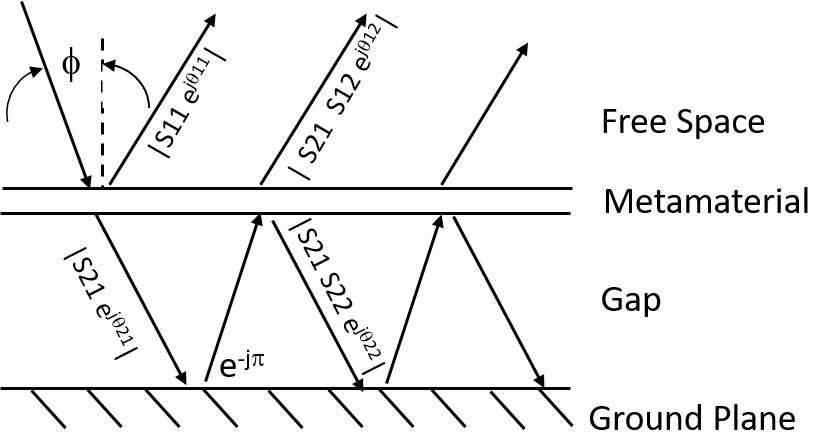

Supplement: Supplementary file 1 — Supplementary Information. [file 41598_2020_66476_MOESM1_ESM.zip › Figures_titled_jpg_3-24-2020/MM-R_interference2.jpg]

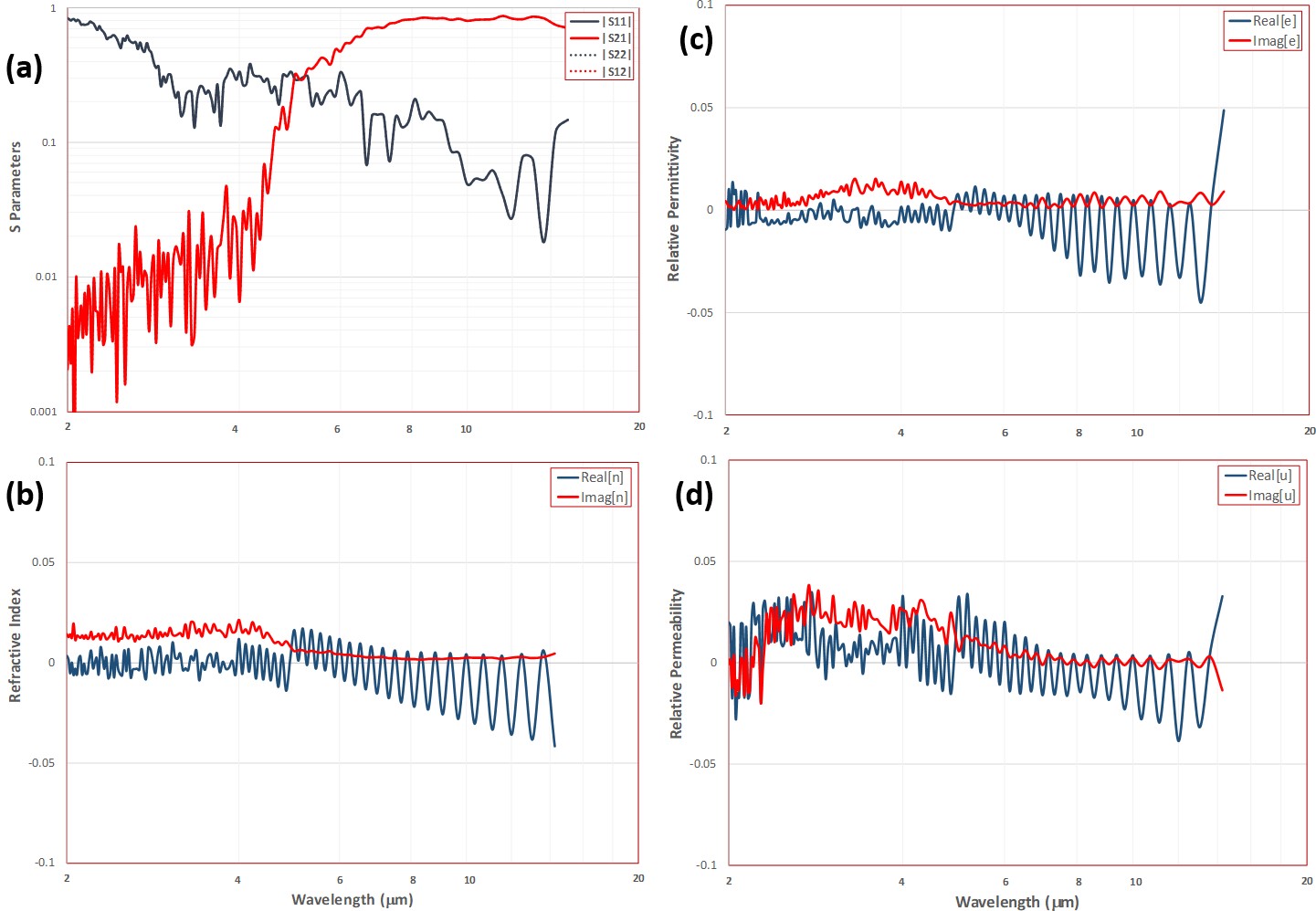

Supplement: Supplementary file 1 — Supplementary Information. [file 41598_2020_66476_MOESM1_ESM.zip › Figures_titled_jpg_3-24-2020/Quad-S-Param_Plot.jpg]

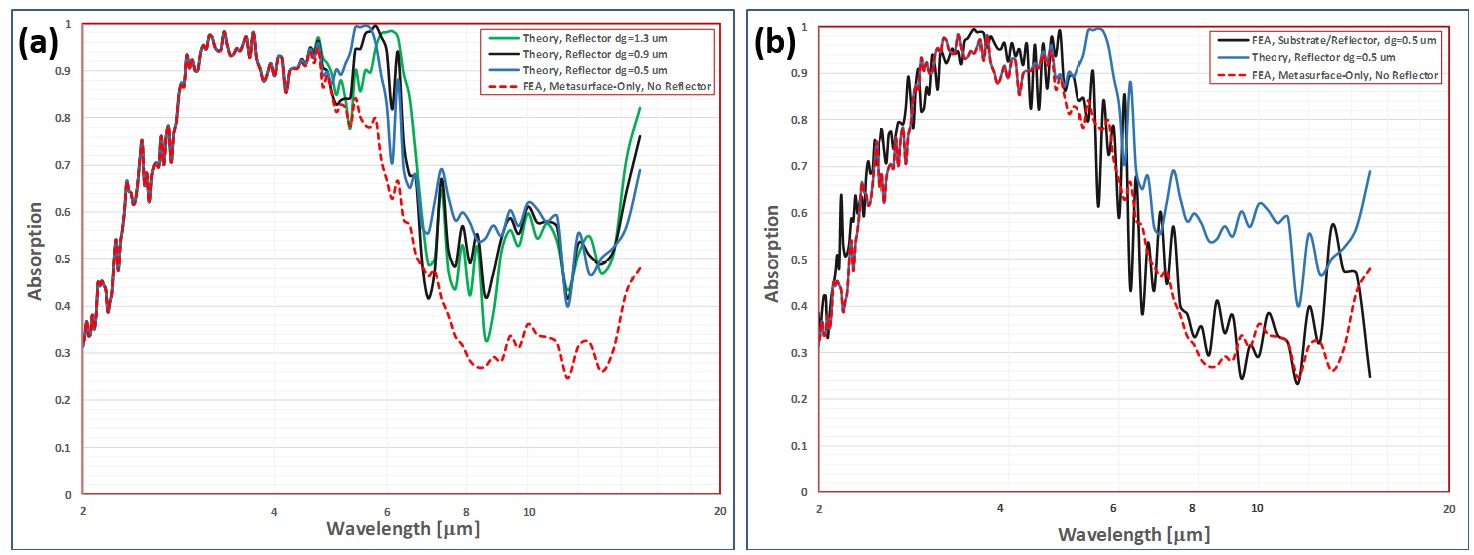

Supplement: Supplementary file 1 — Supplementary Information. [file 41598_2020_66476_MOESM1_ESM.zip › Figures_titled_jpg_3-24-2020/Reflector-d_A-2.jpg]

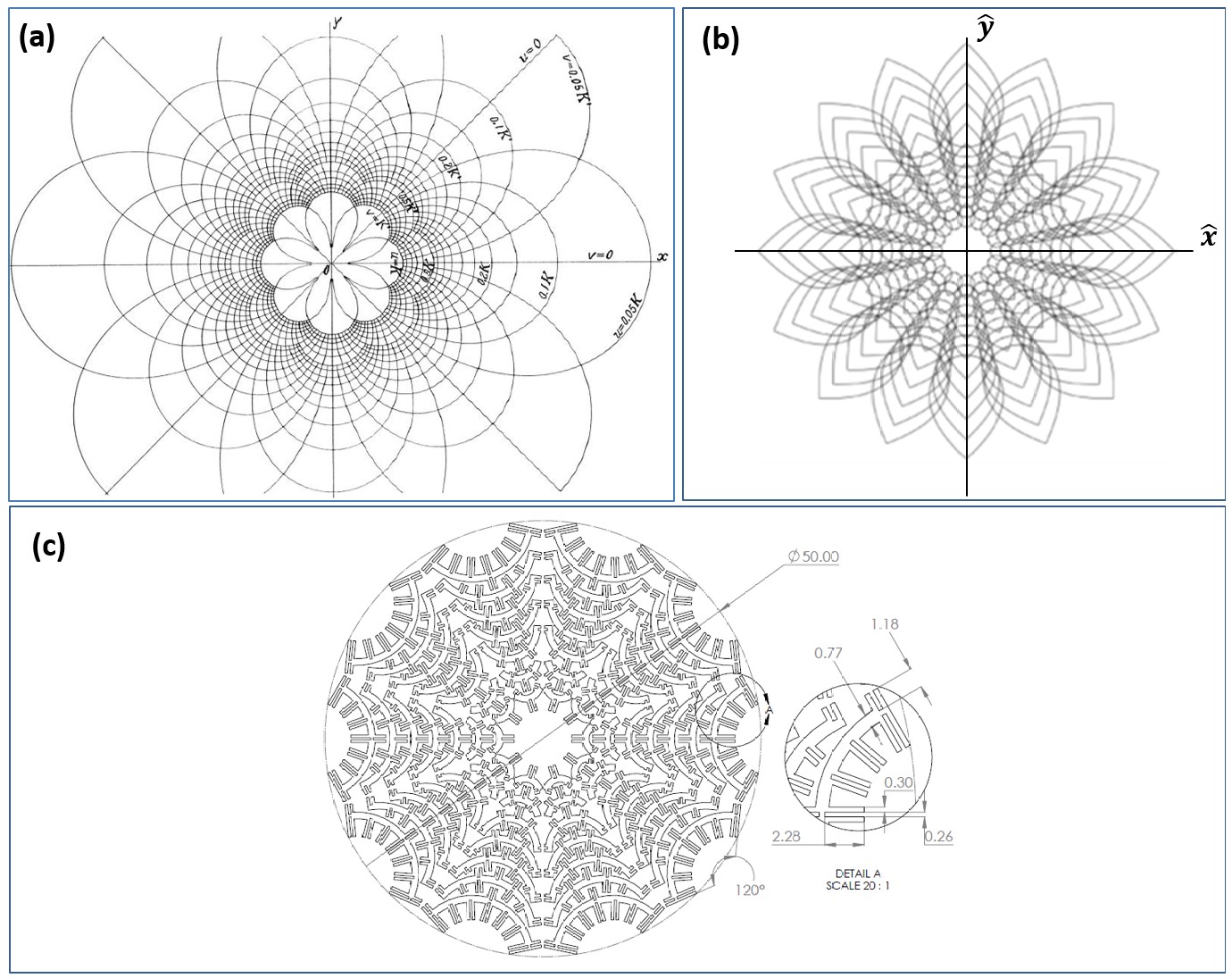

Supplement: Supplementary file 1 — Supplementary Information. [file 41598_2020_66476_MOESM1_ESM.zip › Figures_titled_jpg_3-24-2020/Rhodonea_Inversion_Cell_Description-2.jpg]

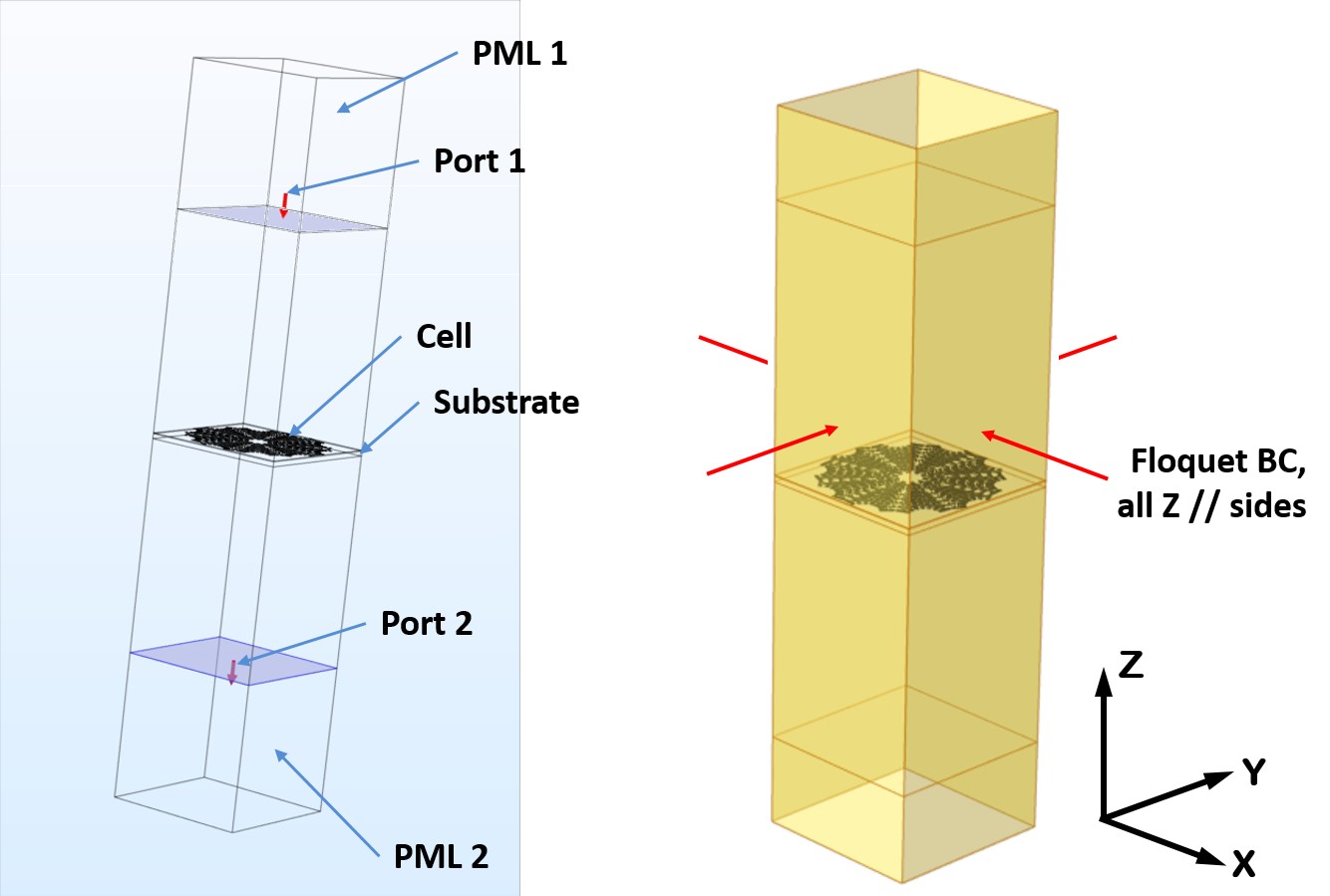

Supplement: Supplementary file 1 — Supplementary Information. [file 41598_2020_66476_MOESM1_ESM.zip › Figures_titled_jpg_3-24-2020/S-Param_FEM.jpg]

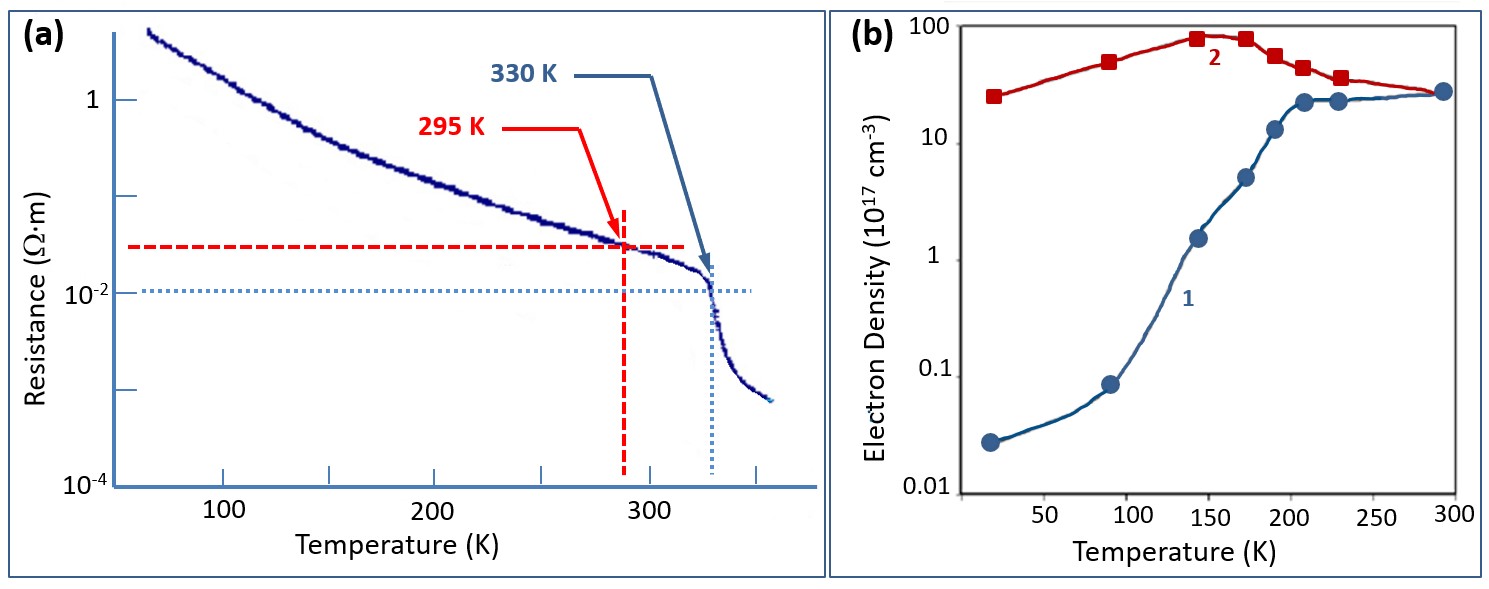

Supplement: Supplementary file 1 — Supplementary Information. [file 41598_2020_66476_MOESM1_ESM.zip › Figures_titled_jpg_3-24-2020/VO2-Properties_Plot-3.jpg]
